# Mechanoecology and Chemoecology: Physical and Chemical Interactions between Insects and Plants

**DOI:** 10.3390/insects14070657

**Published:** 2023-07-23

**Authors:** Gianandrea Salerno, Manuela Rebora, Stanislav Gorb

**Affiliations:** 1 Dipartimento di Scienze Agrarie, Alimentari e Ambientali, University of Perugia, Borgo XX Giugno, 06121 Perugia, Italy; 2 Dipartimento di Chimica, Biologia e Biotecnologie, University of Perugia, Via Elce di Sotto 8, 06121 Perugia, Italy; 3 Department of Functional Morphology and Biomechanics, Zoological Institute, Kiel University, Am Botanischen Garten 9, 24098 Kiel, Germany

Plants and herbivorous insects, as well as their natural enemies such as predatory and parasitoid insects, are united by intricate relationships. During the long period of co-evolution with insects, plants developed a wide diversity of chemical and mechanical features for defense against herbivores, and attracting pollinators and natural herbivore enemies. The chemical basis of insect–plant interactions has been established, and in many examples the feeding and oviposition site selection of phytophagous insects are dependent on plant secondary metabolites. Volatile organic compound (VOC) emission by plants, influenced by insect feeding or oviposition, can repel herbivores and attract natural enemies. In this context, phytophagous and entomophagous insects evolved a finely tuned sensory system for the detection of plant cues.

Despite being often overlooked, mechanical interactions between insects and plants can be rather crucial in the process of host plant selection by phytophagous insects. The evolution of plant surfaces and insect adhesive pads is an interesting example of competition between insect attachment systems and plant anti-attachment surfaces. To achieve sufficient attachment that enables locomotion on widely diverse plant surfaces, insects have evolved various types of leg attachment devices, allowing them to overcome physical plant defenses such as cuticular microfolds, various kinds of trichomes and crystalline wax coverage. Additionally, insect mouthpart mechanics are adapted to certain mechanical properties of the plant surface, which, in combination, may influence insect–plant interactions.

This Special Issue focuses on the chemical and physical interactions between insects and plants in order to understand the functional significance of plant chemistry, plant surface structures and their relationships with a broad range of ecological groups of insects, including pollinators, herbivores and predators.

Dealing with the mechanical interaction between oligophagous phytophagous insect pests and host plant mechanical barriers, Rebora et al. [1] show that olive fruit fly adhesion is reduced by epicuticular waxes on the olive surface, and that the female shows a different ability to attach to the olive surface of different cultivars of *Olea europaea*, in relation to different values of olive surface wettability. On the other hand, Saitta et al. [2] reveal that Cucurbitaceae glandular trichomes do not affect insect attachment ability of the melon ladybird at adult and larval stages, suggesting some adaptation of this insect species to its host plants; moreover, the authors demonstrate that non-glandular trichomes heavily reduce the attachment ability of adults and larvae only when they are dense, short and flexible. In an insect group, such as Phasmatodea, which is highly associated with plants through herbivory and camouflage, Burack et al. [3] reveal that wax crystal-covered plant substrates with fine roughness cause the lowest attachment ability; whereas, strongly structured natural substrates show the highest attachment ability. Gorb and Gorb [4] highlight the contribution of wax coverage on flower stems to impeding the locomotion of the generalist ant species, *Lasius niger*, and provide further evidence for the hypothesis that when having a diversity of plant stems in the field, generalist ants prefer substrates where their locomotion is less hindered by obstacles and/or surface slipperiness. In another study on the interaction between the mechanical features of flowers and the attachment ability of generalist insect pollinators [5], the same authors observe that insect adhesion is surprisingly reduced for petals, where the color intensity is enhanced due to papillate epidermal cells covered by cuticular folds which contribute to adhesion reduction in generalist insect pollinators.

The influence of pollen properties, insect and floral surfaces on the adhesion forces that mediate pollen transfer has been poorly studied thus far; the paper by Huth et al. [6] makes a contribution to this topic, by reporting on the adhesive properties of pollen related to its aging time.

An important aspect of insect–plant interactions is the multitrophic relationships between plants, pests, and natural enemies. In this regard, Farina et al. [7] analyze the damage to plants caused by the cotton whitefly, *Bemisia tabaci*, in presence of a predator which feeds on both insect prey and plant tissue, while Liu et al. [8] study the effects of herbivore-induced rice volatiles and reveal their positive effects on spider attraction and predation ability, with beneficial effects in improving the control of rice pests.

Plant responses to phytophagous insects are extremely complex because of the presence of various types of interactions between plants and pests. In this context, Gao et al. [9] investigate the interplay between insect venom and wounding stress, and the specific expression genes and transcription factors of the Mongolian pine *Pinus sylvestris* var. mongolica. The authors find that insect venom induces a series of physiological changes in the host that weaken the host’s defense response, and hence contribute to the growth of insect symbiotic fungus and the development of eggs.

The use of resistant cultivars is an efficient management strategy against insect pests. Mortazavi Malekshah et al. [10] show that the physicochemical properties of sugarcane cultivars significantly affect *Sesamia nonagrioides* oviposition behavior, life history and population parameters, and recommend a resistant cultivar to reduce damage caused by this pest.

Jakubska-Busse et al. [11] analyze the VOCs emitted by the flowers of an invasive alien plant species and show a list of potential pollinators, in order to shed light on factors enabling the species to rapidly expand.

Changes during leaf ontogeny affect the palatability of insect herbivores, and Lirette et al. [12] summarize the literature describing how chemical defenses of foliage change during the growing season in white spruce, an economically important conifer tree attacked by the eastern spruce budworm *Choristoneura fumiferana*, a specialist growing conifer leaf and bud feeder.

In conclusion, insects and plants have been interacting for more than 350 million years, often resulting in species variability and radiation. Studying insect–plant chemical and mechanical interactions at different levels can help shed light on the complex factors driving the evolutionarily successful relationship between these two groups. Moreover, the results of such investigations can help develop helpful management strategies to successfully control insect pest infestations in cropping environments.
